# Fast neonicotinoid quantification in honey using the one-point internal calibration approach

**DOI:** 10.1016/j.fochx.2024.101565

**Published:** 2024-06-17

**Authors:** Gioele Visconti, Miguel de Figueiredo, Joanie Monnier, Julia Shea, Serge Rudaz, Gaetan Glauser

**Affiliations:** a School of Pharmaceutical Sciences, University of Geneva, CMU – Rue Michel-Servet 1, Geneva, Switzerland; b Institute of Pharmaceutical Sciences of Western Switzerland, University of Geneva, CMU – Rue Michel-Servet 1, Geneva, Switzerland; c Neuchâtel Platform of Analytical Chemistry (NPAC), University of Neuchâtel, Avenue de Bellevaux 51, 2000 Neuchâtel, Switzerland

**Keywords:** Quantification, Internal calibration, LC-MS, Pesticides, Honey

## Abstract

Neonicotinoids, a highly effective class of insecticides used worldwide, have been identified as a major cause of concern for biodiversity. To assess the ecological and environmental consequences of neonicotinoids' use, reliable analytical methodologies, including calibration approaches, are needed. Here, we compared the performance of internal calibration (IC) using a single concentration of stable isotope-labeled standard (SIL) with classical multipoint external calibration (EC) for the quantification of six neonicotinoids in honey. IC showed acceptable levels of trueness (86.3% - 116.0%) and precision (1.4% - 20.8%), although slight biases were observed at very low concentrations compared to EC. When applied to 32 original honey samples, both approaches showed strong agreement (R^2^ > 0.998) with proportional biases lower than 5%. These results highlight the possibility of implementing IC to simplify quantification in liquid chromatography-mass spectrometry-based pesticide applications.

## Highlights

• Stable isotope-labeled internal standards were used as surrogate calibrants.
• The single-point internal calibration (IC) approach was compared with multilevel matrix-matched external calibration.
• Trueness and precision of both approaches were comparable.
• The method was applied to 32 honey samples from Switzerland.
• IC significantly reduces analysis time and instrument turnaround.

## 1 Introduction

In recent years, the use of pesticides in intensive agriculture has become unavoidable in order to ensure sufficient food production for an ever-growing population (Baynes et al., 2016; Carvalho, 2017). Since their introduction in 1990, neonicotinoids have become the most widely used class of insecticides, replacing earlier pesticides such as organophosphates and carbamates (Jeschke, Nauen, Schindler, & Elbert, 2011). Neonicotinoids are systemic insecticides acting as agonists of the nicotinic acetylcholine receptors of insects (Bonmatin et al., 2015; Simon-Delso et al., 2015). Due to their systemic accumulation in the treated plants and long persistence in the environment, neonicotinoids represent a threat for non-target insects, including beneficial pollinators such as honeybees and wild bees (Goulson, 2013). At field-realistic concentrations, neonicotinoids are usually not directly lethal, but may still harm bees and other non-target insects by causing sublethal effects, such as neurological and cognitive disorders, reduced foraging and homing ability, immunosuppression, or reproductive effectiveness (Pisa et al., 2021). A 2017 study by Mitchell and colleagues showed that nearly half of the world's honey contained levels of neonicotinoids >0.1 ng/g, a concentration which is potentially harmful to bees (Mitchell et al., 2017). By contrast, neonicotinoid residues have rarely been found in the bees themselves, likely because of the fast metabolism of these molecules (Blacquière, Smagghe, van Gestel, & Mommaerts, 2012). Following the massive and global use of neonicotinoids for more than two decades, the scientific community has repeatedly raised concerns about the risks they pose to biodiversity, which eventually resulted in a partial ban in the European Union in 2018 (Woodcock et al., 2018). Neonicotinoids are, nevertheless, still used without restriction in most regions of the world.

The possible presence of neonicotinoid residues in food and environmental matrices has raised the need for selective and sensitive analytical techniques which can detect and quantify them at trace levels (Wang et al., 2022). In this context, the vast majority of studies has reported the use of (ultra-)high-performance liquid chromatography coupled to tandem mass spectrometry ((U)HPLC–MS/MS) for neonicotinoid quantification (Hou et al., 2023; Kamel, 2010; Kammoun, Mulhauser, Aebi, Mitchell, & Glauser, 2019; Valverde, Ares, Bernal, Nozal, & Bernal, 2022; Yáñez, Martín, Bernal, Nozal, & Bernal, 2014). The impact of the calibration approach on the quantitative performance has, however, largely been overlooked. To obtain true and precise results, the calibration methodology should be carefully investigated (Lehotay, Mastovska, Lightfield, & Gates, 2010; Zrostlíková, Hajšlová, Poustka, & Begany, 2002. Multipoint external calibration (EC) is the gold standard in international guidelines for the validation of analytical methods. With EC, the composition of the calibration standards should be as similar as possible to that of the samples, a procedure referred to as matrix-matched calibration (Visconti et al., 2023). To compensate for differences in matrix effects and extraction recovery between the calibration standards and the study samples, internal standards are usually added before sample preparation (Cuadros-Rodríguez, Bagur-González, Sánchez-Viñas, González-Casado, & Gómez-Sáez, 2007; Wu, Dong, Mei, Ning, & She, 2020). It is generally accepted that stable isotope-labeled (SIL) analogs represent the best internal standards due to their similar physicochemical properties to the analytes of interest (Stokvis, Rosing, & Beijnen, 2005).

When using the EC approach, the preparation of calibration curves can be time-consuming and prone to errors, particularly with multi-residue methods (Sun et al., 2023). Recently, the use of SILs as surrogate calibrants has been proposed to simplify and accelerate the process (Aydin, Drotleff, Noack, Derntl, & Lämmerhofer, 2021; Banno & Yabuki, 2020; Rule & Rockwood, 2016). When a single level of SIL is added to the sample and analyzed as a whole together with the analyte, the process is referred to as internal calibration (IC) because the sample standardization and calibration are performed directly in the study sample (Cuadros-Rodríguez et al., 2007). There are clear advantages in using IC over EC: analytical workflows are simplified and the absence of analyte-free matrices is no longer an issue (Cuadros-Rodríguez et al., 2007; Visconti et al., 2023). Moreover, using the SIL as an internal calibrator directly in the sample best corrects not only for recovery but also for sample-specific matrix effect, thus for overall analytical variation. Several research groups have demonstrated that IC can show comparable performance to EC in bioanalysis (Gu et al., 2019; Visconti et al., 2023; Yuan, Gu, Zeng, Pillutla, & Ji, 2020), but in the field of environmental analysis, the approach is just emerging (Zhang et al., 2023).

In this work, we evaluated the one-point IC as a potential approach for the quantification of six neonicotinoids in honey by UHPLC–MS/MS. To do so, a validation dataset was processed with both EC and IC and their performance compared in terms of precision, trueness and coverage. In a second step, we measured the concentrations of neonicotinoids in 32 original honey samples from the Neuchâtel region of Switzerland to illustrate the applicability of IC.

## 2 Experimental

### 2.1 Dataset used

**Fig. 1:**
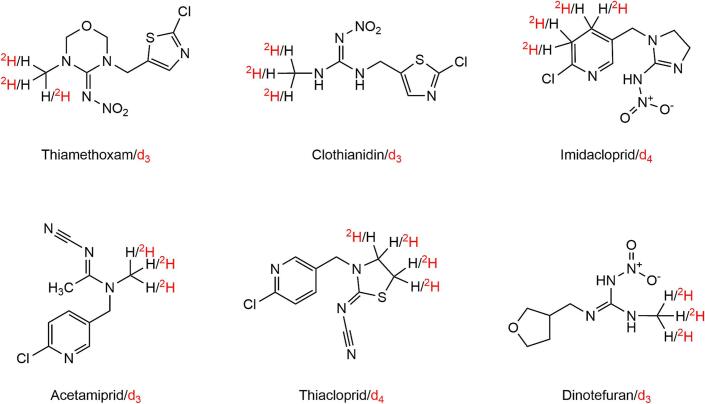
Structures of the neonicotinoids and their respective stable isotope labeled standard (in red) used in this study. (For interpretation of the references to colour in this figure legend, the reader is referred to the web version of this article.)

For systematic evaluation of EC and IC, a complete dataset from a previously published study in which method validation by EC had been performed according to international guidelines was first used (Kammoun et al., 2019). Six major neonicotinoids (thiamethoxam, clothianidin, imidacloprid, acetamiprid, thiacloprid, and dinotefuran) were targeted and quantification was performed using their corresponding deuterated SIL analogs (Fig. 1).

In addition, an original dataset consisting of 32 spring honey samples from the Neuchâtel region (Switzerland) collected between 2020 and 2021 was used to further compare the quantitative performance between the IC and the EC. To obtain honey samples, the Neuchâtel canton was first separated in 4 km^2^ sectors and all known beehives in the sectors identified. Beekeepers were then contacted to provide their honey on a voluntary basis. Out of 45 requests, 32 samples collected at elevations ranging from 431 to 1143 m above sea level were received.

### 2.2 Extraction and analysis of honey samples

All honey samples were prepared according to Kammoun et al. (Kammoun et al., 2019). Briefly, 10 mL of water:acetonitrile (50:50) and 20 μL of internal standard solution (125 ng/mL) were added to 0.5 g of honey. 2 g of magnesium sulfate, 0.5 g of sodium chloride, 0.5 g of sodium citrate dihydrate, and 0.25 g of sodium citrate sesquihydrate were added, the tubes were shaken and centrifuged at 4000*g* for 5 min. The epiphase was collected and 0.15 g of magnesium sulfate, 0.1 g C18 and 0.1 g PSA bulk phases were added for solid phase dispersive extraction. After homogenization and centrifugation, the supernatant was collected, evaporated, resuspended in 0.5 mL MeOH 25%, and filtered through hydrophilic PTFE syringe filters prior to UHPLC-MS/MS injection.

The samples from the first dataset were measured using the exact same procedure described in Kammoun et al. (Kammoun et al., 2019). Briefly, the analysis was performed on a Cortecs C18+ column (2.1 × 100 mm, 1.6 μm) using an Acquity UPLC (Waters Corp.) coupled to a TQ-S triple quadrupole (Waters Corp.). A binary solvent mixture composed of water, 0.05% formic acid and 5 mM ammonium formate as mobile phase A and acetonitrile, 0.05% formic acid and 5 mM ammonium formate as mobile phase B was used in gradient mode. The samples from the second dataset were analyzed using the same method but on a slightly modified analytical system consisting of an Acquity I-Class system (Waters Corp.) coupled to a TQ-XS triple quadrupole (Waters Corp.).

### 2.3 Validation and comparison of EC and IC approaches

Method validation was performed according to SANTE/11813/ 2017 guidelines (European Commission, 2017). External calibration curves were generated at seven concentration levels using honey samples without any quantifiable traces of neonicotinoids as blank matrix (#JBN313, JBN#446 and JBN#515). Trueness and repeatability were determined at four different concentrations using five quality control replicates for each concentration level (Table S1). Intra-day precision (i.e. repeatability) was expressed as the coefficient of variation (CV%). Trueness was calculated as the average of the relative bias obtained for each concentration level. The method was considered accurate if the precision was below 20% and trueness was in the range 70–120% (European Commission, 2017).

The linearity range and concentration coverage of the IC approach in comparison to the EC were determined as follows: for the linearity range, the lower and upper limits of quantification were established based on accuracy profiles as the concentrations at which the total error (i.e., the combination of bias and precision) exceeded 30%. Concentration coverage was then calculated as the ratio of IC and EC linearity ranges in which 95% of the results fell within 70% and 120% (Visconti et al., 2023).

### 2.4 Data treatment and statistical analysis

TargetLynx 4.2 (Waters Corporation, Milford, MA, USA) was used to automatically integrate peak areas, which were subsequently manually checked. One-way ANOVA was performed with MedCalc version 19.7.2 (MedCalc Software, Ostend, Belgium). GLM with Poisson distribution was conducted in R version 4.3.3. All calculations for the IC methodology including the determination of the response factors (RF), concentrations of analytes in the validation and study samples, and Passing-Bablok regressions were performed in Python 3.9 using an automated in-house pipeline described elsewhere (Visconti et al., 2023) and available upon request. For each analyte, RFs were calculated at three concentrations (10 pg/mL, 100 pg/mL and 10,000 pg/mL) and over three days. An average RF was determined from these nine values.

## 3 Results and discussion

To compare EC and IC approaches for the quantification of 6 neonicotinoids in honey, a validation dataset from a published method was used (Kammoun et al., 2019). In the original EC approach, neonicotinoid concentrations were measured using an analyte-free honey sample spiked with isotope-labeled (SIL) analogs as internal standards (Kammoun et al., 2019). For IC, the role of the SIL was modified to that of a surrogate calibrant and the MS response of the analyte was translated into a concentration through the analyte-to-SIL ratio (Visconti et al., 2023).

### 3.1 Validation dataset

First, an average RF was determined for each neonicotinoid (Table 1). The validation data were then evaluated in terms of repeatability, trueness, linearity range and concentration coverage for both EC and IC (Table 1). Overall, repeatability and trueness were similar between both approaches, and although some neonicotinoids had a slightly higher lower limit of quantification (LLOQ) with IC, the concentration coverage exceeded 99% (Table 1, Table S2 and S3). More specifically, trueness values for thiamethoxam, clothianidin and imidacloprid were within the 70–120% limits at all concentrations tested, thus linearity ranges between EC and IC were identical and coverage was 100%. For acetamiprid, thiacloprid and dinotefuran, slight biases were observed at the lowest concentration values (conc. 1). For example, acetamiprid and thiacloprid showed a positive bias of 147.7% (+0.95 pg/g) and 127.7% (+0.55 pg/g), respectively, at the nominal value of 2 pg/g. Dinotefuran showed a negative bias of 58.6% (−8.28 pg/g) at the nominal value of 20 pg/g. Several hypotheses could explain the discrepancies between EC and IC: first some SIL chemical impurities might contribute to the analyte MS/MS signal or, alternatively, some isotopic contributions from the analyte might contribute to the SIL MS/MS signal as demonstrated by Castillo et al. (Castillo et al., 2013); then the analyte signal might be suppressed by SIL's competition in the electrospray process (Khamis, Adamko, & El-Aneed, 2021); lastly the RF might drift over days. (Jones, Schultz, Eckstein, & Ackermann, 2012). Crosstalk contributions were evaluated by injecting the SIL analog individual solutions at 5 ng/mL and checking the MS/MS channel of the analytes. No crosstalk was observed for any of the analytes. By contrast, isotopic contributions of the analytes in the SILs MRM transitions were clearly present. For instance, when the main transition (295 *m/z* > 211 *m/z*) of the SIL was used, interferences up to 0.52% were observed for thiamethoxam (Table S4). This isotopic contribution could be reduced to 0.03% by using a suboptimal transition, such as 295 *m/z* > 132 *m/z* initially considered as a qualifier (Table S4). The isotopic contribution would be particularly relevant when the analyte concentration in the study sample is markedly higher than that of the SIL. Using SILs with more deuterium atoms, if available, may also reduce the risk of interferences, albeit at the possible expense of retention time shifts. When several SILs are available, the use of an isotopic distribution calculator will help select the most appropriate labeling for the SIL (Fig. S1).

**Table 1:** Comparison of quantitative performances between the external (EC) and internal (IC) calibration approaches. The $\bar{\mathrm{RF}}\pm \mathrm{SD}$ was calculated over three days. SIL: stable isotope-labeled, RF: response factor.

#				EC				IC			
	Analyte	SIL analog	$\bar{\mathrm{RF}}\pm \mathrm{SD}$	Trueness (%)	Repeatability (%)	Linearity range (pg⋅g^−1^)		Trueness (%)	Repeatability (%)	Linearity range (13 ng⋅g^−1^)	Coverage (%)
1	Dinotefuran	-d3	1.080 ± 0.006	93.2–111.5	1.6–13.3	20–10′000		58.6–89.8	1.7–12.9	50–10′000	99.70
2	Thiamethoxam	-d3	1.007 ± 0.014	103–113.2	1.5–8.3	5–10′000		98.1–106.7	1.5–10.9	5–10′000	100
3	Clothianidin	-d3	1.237 ± 0.013	98.7–112.3	4–9.1	10–10′000		97.7–99.5	4–10.3	10–10′000	100
4	Imidacloprid	-d4	1.037 ± 0.004	100.8–115.2	1.6–10.8	10–10′000		101.9–115	1.6–10.6	10–10′000	100
5	Acetamiprid	-d3	1.070 ± 0.024	105.7–116	1.5–9.3	2–10′000		98.4–147.7	1.6–6.5	5–10′000	99.97
6	Thiacloprid	-d4	1.009 ± 0.059	106.9–112.1	1.4–22.3	2–10′000		101.4–125.7	1.4–20.8	5–10′000	99.97

**Fig. 2:**
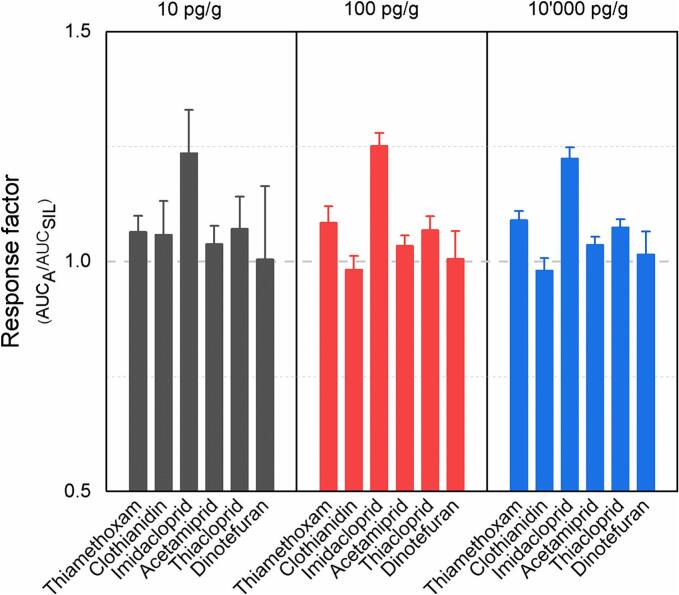
Response factor mean and standard deviation values at three different concentrations over three days. A: analyte, SIL: stable isotope-labeled standard.

The second hypothesis is related to ionization competition that may occur between the SIL, the analyte and coeluting compounds in the MS source (Enke, 1997). The ionization ratios were thus evaluated by analyzing different mixtures with identical concentrations of both SILs and analytes in neat solutions. Overall, the RF coefficient of variation was below 15% over the three days for all the neonicotinoids (Fig. 2). Analyte to SIL ratios were close to 1 over the dynamic range studied, except for imidacloprid which presented a ratio of 1.25. The ratios remained similar using spiked honey samples (Table S5), which suggests a negligible impact of the matrix on the compound ionization ratios. This observation is valid for honey but may not apply to other foodstuffs (e.g. green onion) as Nakamura and colleagues found that deuterated neonicotinoid standards do not always correct for matrix effects in an optimal manner (Nakamura, Otake, Hanari, & Takatsu, 2019). In summary, the obtained analyte-to-SIL ratios confirmed the possibility of using the SIL as an internal surrogate calibrant.

### 3.2 Application to honey samples

**Fig. 3:**
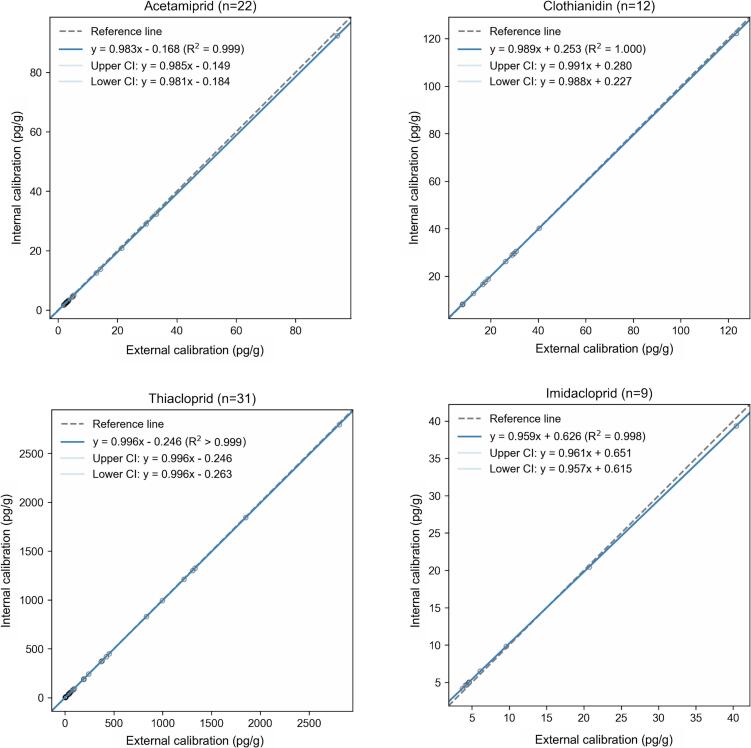
Passing-Bablok regression plots for the four detected neonicotinoids in honey using external versus internal calibration. Blue lines represent the lines of best fit, grey dashed lines represent the lines of identity, and light blue areas indicate the 95% confidence intervals. R^2^, determination coefficient, CI confidence interval. (For interpretation of the references to colour in this figure legend, the reader is referred to the web version of this article.)

To illustrate the applicability of IC, the six neonicotinoids were measured in 32 individual honey samples from the Neuchâtel region, and the inverse-predicted concentrations were compared with those measured with the EC approach using the Passing-Bablok methodology. Regression plots were assessed for each analyte and excellent coefficients of determination (R^2^ > 0.998) were obtained between IC and EC for the four detected compounds (Fig. 3). Acetamiprid, clothianidin, thiacloprid and imidacloprid showed an average proportional bias of −4.1%. Moreover, the intercept values were very close to zero, which provides further evidence that IC may replace EC not only at high but also at low concentration levels. Dinotefuran and thiamethoxam were below LLOQ in all samples.

Overall, 96% and 91% of the samples contained at least one neonicotinoid at concentrations above LLOD and LLOQ, respectively. Two neonicotinoids were detected in 43% of the samples, and 38% contained three to four neonicotinoids. Interestingly, the only sample with no detectable traces of any neonicotinoid was that from the hive at the highest altitude (1143 m.a.s.l.), and a significant negative correlation between the total concentration of pesticides and elevation was observed (GLM with Poisson distribution; regression coefficient = −0.003, z value with 30 df = 2.32, *p* = 0.02). The total concentration of all neonicotinoids was 0.41 ng/g on average and the maximal concentration in an individual sample was 2.72 ng/g. Maximum residue levels were not reached for any of the pesticides.

## 4 Conclusion and perspectives

In this study, a calibration design initially made for multi-level EC was retrospectively used for an IC and both approaches were compared in terms of quantification performance. The results show that the one-point IC performs similarly to the EC approach and is robust enough to allow the quantification of neonicotinoids over a wide concentration range in honey samples, although slight biases may be observed at concentrations close to LLOQ. After determining response factors, IC eliminates the need for an EC curve for each batch of samples and becomes significantly faster in routine. It remains to be seen whether IC can be reliably applied to other, potentially more complex food matrices than honey.

## Funding

This research was funded by the University of Neuchâtel and the University of Geneva.

## CRediT authorship contribution statement

**Gioele Visconti:** Writing – review & editing, Writing – original draft, Methodology, Investigation, Formal analysis, Conceptualization. **Miguel de Figueiredo:** Writing – review & editing, Investigation, Formal analysis. **Joanie Monnier:** Investigation, Formal analysis. **Julia Shea:** Investigation, Formal analysis. **Serge Rudaz:** Writing – review & editing, Supervision, Resources, Methodology, Conceptualization. **Gaetan Glauser:** Writing – review & editing, Writing – original draft, Supervision, Resources, Conceptualization.

## Declaration of competing interest

The authors declare that they have no known competing financial interests or personal relationships that could have influence the work reported in this paper.

## Data Availability

Data will be made available on request.

## Appendix A Supplementary data

Supplementary information Image 1
