# Supplementary material for: Fast neonicotinoid quantification in honey using the one-point internal calibration approach
Source: Food Chem X. 2024 Jun 17;23:101565. doi: 10.1016/j.fochx.2024.101565 (PMC11239451; doi:10.1016/j.fochx.2024.101565)

**SUPPLEMENTARY INFORMATION**

**Fast neonicotinoid quantification in honey using the one-point internal calibration approach**

Gioele Visconti^1,2^, Miguel de Figueiredo^1,2^, Joanie Monnier^1,2,3^, Julia Shea^3^, Serge Rudaz^1,2^, Gaetan Glauser^3^

1. School of Pharmaceutical Sciences, University of Geneva, CMU – Rue Michel-Servet 1, Geneva, Switzerland.
2. Institute of Pharmaceutical Sciences of Western Switzerland, University of Geneva, CMU – Rue Michel-Servet 1, Geneva, Switzerland.
3. Neuchâtel Platform of Analytical Chemistry (NPAC), University of Neuchâtel, Avenue de Bellevaux 51, 2000 Neuchâtel, Switzerland.

**Correspondence**

Gaetan Glauser, Neuchâtel Platform of Analytical Chemistry (NPAC), Université de Neuchâtel, Avenue de Bellevaux 51, 2000 Neuchâtel, Switzerland

E-mail: [gaetan.glauser@unine.ch](mailto:gaetan.glauser@unine.ch)

**Table S1**. Concentrations of analytes used for the validation.

| **Analyte** | **Conc. 1 (pg/g)** | **Conc. 2 (pg/g)** | **Conc. 3 (pg/g)** | **Conc. 4 (pg/g)** |
| --- | --- | --- | --- | --- |
| Dinotefuran | 20 | 50 | 500 | 10000 |
| Thiamethoxam | 5 | 10 | 500 | 10000 |
| Clothianidin | 10 | 20 | 500 | 10000 |
| Imidacloprid | 10 | 20 | 500 | 10000 |
| Acetamiprid | 2 | 5 | 500 | 10000 |
| Thiacloprid | 2 | 5 | 500 | 10000 |

**Table S2.** Repeatability and trueness of the method using the external (EC) calibration approach.

|  | **Repeatability (%RSD, n=5)** | | | |  | **Trueness (%, n=5)** | |  |  |
| --- | --- | --- | --- | --- | --- | --- | --- | --- | --- |
| **Analyte** | **Conc. 1** | **Conc. 2** | **Conc. 3** | **Conc. 4** |  | **Conc. 1** | **Conc. 2** | **Conc. 3** | **Conc. 4** |
| Dinotefuran | 5.0 | 13.3 | 1.6 | 4.7 |  | 93.2 | 111.5 | 106.4 | 110.6 |
| Thiamethoxam | 8.3 | 7.1 | 1.5 | 3.8 |  | 113.2 | 103.0 | 108.3 | 106.4 |
| Clothianidin | 9.1 | 7.1 | 4.0 | 4.1 |  | 112.3 | 105.0 | 98.7 | 105.5 |
| Imidacloprid | 5.7 | 10.8 | 3.6 | 1.6 |  | 112.3 | 100.8 | 115.2 | 110.6 |
| Acetamiprid | 9.3 | 7.2 | 1.5 | 4.7 |  | 116.0 | 109.6 | 108.0 | 105.7 |
| Thiacloprid | 7.5 | 22.3 | 1.4 | 4.1 |  | 112.0 | 111.6 | 106.9 | 112.1 |

**Table S3.** Repeatability and trueness of the method using the internal (IC) calibration approach.

|  | **Repeatability (%RSD, n=5)** | | | |  | **Trueness (%, n=5)** | |  |  |
| --- | --- | --- | --- | --- | --- | --- | --- | --- | --- |
| **Analyte** | **Conc. 1** | **Conc. 2** | **Conc. 3** | **Conc. 4** |  | **Conc. 1** | **Conc. 2** | **Conc. 3** | **Conc. 4** |
| Dinotefuran | 6.5 | 12.9 | 1.7 | 4.7 |  | 58.6 | 86.3 | 89.8 | 110.4 |
| Thiamethoxam | 10.9 | 7.1 | 1.5 | 3.9 |  | 106.7 | 98.1 | 104.0 | 107.3 |
| Clothianidin | 10.3 | 7.5 | 4.0 | 4.1 |  | 99.5 | 98.2 | 97.7 | 101.9 |
| Imidacloprid | 5.2 | 10.6 | 3.6 | 1.6 |  | 115.0 | 101.9 | 114.7 | 111.9 |
| Acetamiprid | 6.5 | 6.4 | 1.6 | 4.7 |  | 147.7 | 116.0 | 103.3 | 98.4 |
| Thiacloprid | 6.2 | 20.8 | 1.4 | 4.1 |  | 125.7 | 113.4 | 101.4 | 104.4 |

**Table S4.** Isotopic contribution evaluation coming from the analyte on the SIL analog. N/A: not applicable.

| **Ion isotope** | **MS/MS transition *(m/z)*** | **Contribution (%)** |
| --- | --- | --- |
| Thiamethoxam | | |
| *M* | *292 > 211* | N/A |
| M+3 | 295 > 214 | 0.52 |
| M+3 | 295 > 184 | 0.19 |
| M+3 | 295 > 132 | 0.03 |
| Clothianidin | | |
| *M* | *250 > 169* | N/A |
| M+3 | 253 > 172 | 0.37 |
| M+3 | 253 > 132 | 0.02 |
| Imidacloprid | | |
| *M* | *256 > 209* | N/A |
| M+3 | 260 > 213 | 0.15 |
| M+3 | 260 > 179 | 0.01 |

**Table S5.** Response factor (RF) comparison between neat solution (MeOH 25%) and neonicotinoids-free honey samples (JBN#446 and JBN#515) for each neonicotinoid. Mean values and standard deviations were measured in triplicate at the concentrations of 10, 100 and 10’000 pg⋅mL^-1^ for each matrix.

| **Analyte** | **RF_neat solution_** | **RF_honey_** | **P-value** |
| --- | --- | --- | --- |
| Thiamethoxam | 1.118 ± 0.013 | 1.116 ± 0.007 | 0.377 |
| Clothianidin | 1.042 ± 0.009 | 1.076 ± 0.041 | 0.051 |
| Imidacloprid | 1.307 ± 0.023 | 1.288 ± 0.050 | 0.269 |
| Acetamiprid | 1.069 ± 0.011 | 1.074 ± 0.021 | 0.331 |
| Thiacloprid | 1.093 ± 0.012 | 1.092 ± 0.027 | 0.478 |
| Dinotefuran | 1.025 ± 0.032 | 1.027 ± 0.051 | 0.453 |

**Figure S1**. Simulated isotopic distribution for the investigated analytes (in black) and their respective SIL (in red).


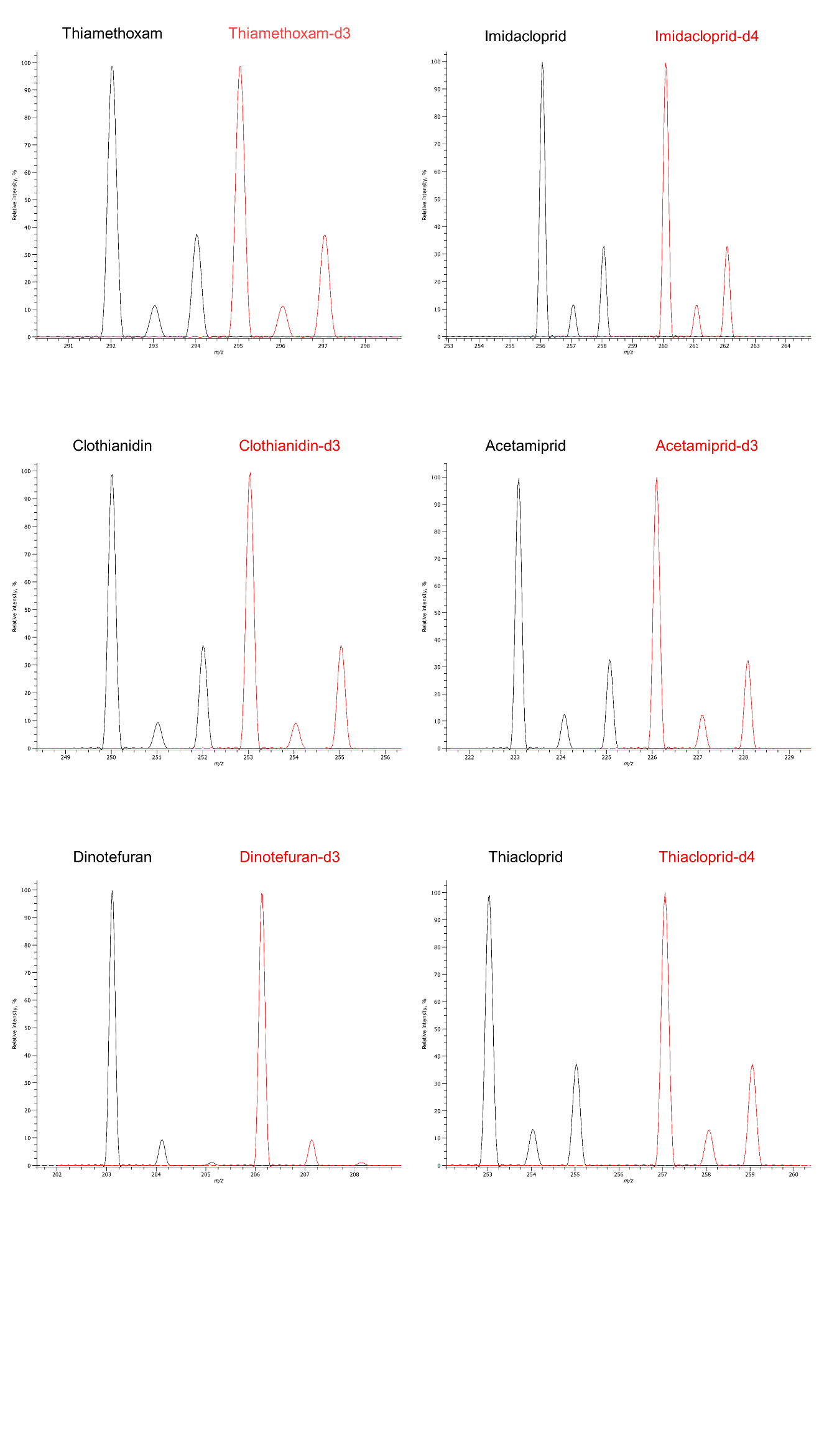

Supplement: Supplementary file 1 — Supplementary information [file mmc1.docx]
